# Post-exercise Warm or Cold Water Immersion to Augment the Cardiometabolic Benefits of Exercise Training: A Proof of Concept Trial

**DOI:** 10.3389/fphys.2021.759240

**Published:** 2021-11-03

**Authors:** Brooke M. Russell, Courtney R. Chang, Terry Hill, James D. Cotter, Monique E. Francois

**Affiliations:** ^1^School of Medicine, University of Wollongong, Wollongong, NSW, Australia; ^2^Illawarra Health and Medical Research Institute, Wollongong, NSW, Australia; ^3^School of Physical Education, Sport and Exercise Sciences, University of Otago, Dunedin, New Zealand

**Keywords:** exercise, metabolic flexibility, glucose, hydrotherapy, water immersion

## Abstract

We investigated whether substituting the final half within 60-min bouts of exercise with passive warm or cold water immersion would provide similar or greater benefits for cardiometabolic health. Thirty healthy participants were randomized to two of three short-term training interventions in a partial crossover (12 sessions over 14–16 days, 4 week washout): (i) EXS: 60 min cycling 70% maximum heart rate (HRmax), (ii) WWI: 30 min cycling then 30 min warm water (38–40°C) immersion, and/or (iii) CWI: 30 min cycling then 30 min cold water (10–12°C) immersion. Before and after, participants completed a 20 min cycle work trial, $\dot{\text{V}}$O_2_max test, and an Oral Glucose Tolerance Test during which indirect calorimetry was used to measure substrate oxidation and metabolic flexibility (slope of fasting to post-prandial carbohydrate oxidation). Data from twenty two participants (25 ± 5 year, BMI 23 ± 3 kg/m^2^, Female = 11) were analyzed using a fixed-effects linear mixed model. $\dot{\text{V}}$O_2_max increased more in EXS (interaction *p* = 0.004) than CWI (95% CI: 1.1, 5.3 mL/kg/min, Cohen’s *d* = 1.35), but not WWI (CI: −0.4, 3.9 mL/kg/min, *d* = 0.72). Work trial distance and power increased 383 ± 223 m and 20 ± 6 W, respectively, without differences between interventions (interaction both *p* > 0.68). WWI lowered post-prandial glucose ∼9% (CI −1.9, −0.5 mmol/L; *d* = 0.63), with no difference between interventions (interaction *p* = 0.469). Substituting the second half of exercise with WWI provides similar cardiometabolic health benefits to time matched exercise, however, substituting with CWI does not.

## Introduction

Physical inactivity has been reported as the fourth leading cause of death from chronic disease worldwide (World Health Organization [WHO], 2009) and is a significant contributor to preventable morbidity and mortality in Australian adults (Australian Bureau of Statistics, 2018). Despite ample evidence supporting the health benefits of physical activity, alarmingly, only 15% of Australian adults meet the “Australian Physical Activity and Sedentary Guidelines” of 150–300 min of moderate intensity physical activity per week (Australian Bureau of Statistics, 2018; Australian Government Department of Health [AGDH], 2020). Alternate strategies that assist and encourage those who are unable and/or unwilling to engage in sufficient levels of physical activity are urgently needed.

Post-exercise water immersion has long been used by athletes in an attempt to improve recovery and to enhance exercise capacity (Versey et al., 2013; Machado et al., 2017; Broatch et al., 2018; Chow et al., 2018; Tavares et al., 2018). Cold water immersion (CWI: 5–16°C) has been shown to stimulate metabolic regulators such as mitochondrial biogenesis (Broatch et al., 2018). For example, Ihsan et al. (2014, 2015) found an increase in skeletal muscle PGC-1α mRNA concentration, an important regulator of mitochondrial biogenesis and oxidative metabolism, when CWI (10°C for 15 min) occurred post-treadmill running. Conversely, prior work by Broatch et al. (2017) showed post-exercise CWI did not improve $\dot{\text{V}}$*O*_2_ max or maximal uncoupled respiration (complexes I and II), compared to 6-weeks of exercise training. Literature is inconsistent, with some studies finding improvements with CWI (reviewed in Broatch et al., 2018), while others observe no additional benefit in mitochondrial function and endurance performance with post-exercise CWI (Shute et al., 2017; Broatch et al., 2018). Whilst the inconsistent findings may be due to various factors, including differences in methodologies and participants’ body composition, it is plausible that the reduction in limb blood flow with CWI, in part, contributes to impairments in vascular function, metabolic flexibility and performance (Yamane et al., 2006, 2015; Stephens et al., 2018).

In contrast to CWI, warm water immersion (WWI: 37–46°C) increases limb blood flow, and muscle heating activates regulators of mitochondrial biogenesis and glucose sensitivity, it thereby offers potential alternate or adjunct to exercise training and is currently being investigated by many researchers for its health and performance benefits. WWI alone has been found to improve fasting glucose, glucose uptake (Gupte et al., 2009; Sjøberg et al., 2017), insulin concentrations (Hoekstra et al., 2018), vascular function and structure (Green et al., 2010; Brunt et al., 2016), and $\dot{\text{V}}$O_2_max, comparably to exercise training (Bailey et al., 2016). The only study to date, Bailey et al. (2016), observed similar increases in cardiorespiratory fitness and cerebrovascular function following 8 weeks of half hourly WWI sessions when compared to time-matched exercise. In addition, just 10 WWI sessions in sedentary, overweight adults or 3 weeks of WWI in type 2 diabetes patients was found to increase nitric oxide availability and reduce fasting insulin and glucose concentrations (Stephens et al., 2018). Collectively, these studies indicate that WWI might be used as a viable substitute in achieving some of the benefits of exercise training (e.g., reduced risk of diabetes, improved heart health, improved cardiovascular fitness) particularly for those who cannot or will not exercise sufficiently. Furthermore, prior research has shown that the combination of passive heat and exercise (rather than complete substitution) results in additive increases in both metabolic enzyme and protein adaptations in the skeletal muscle of mice (Tamura et al., 2014). Together, the key elements of heat, coupled with the mechanical tension, substrate turnover and transient oxidative stress experienced during exercise, drive the wide-ranging and unparalleled health benefits of exercise. Therefore, the combination of WWI and exercise, for those who cannot perform sufficient volumes of exercise, may be more beneficial than WWI alone in achieving positive health outcomes associated with regular exercise (i.e., also including those for bone and muscle). Further, substituting the second half of exercise with WWI may allow those who are disinclined or less able to meet exercise recommendations to achieve comparable health benefits. The present study investigates the efficacy of post-exercise warm or cold WI in an apparently health population before aiming to apply this approach to clinical populations.

To date, no research has examined the chronic cardiometabolic health effects of replacing half the exercise duration with warm or cold water immersion as an alternate strategy to time-matched exercise. Based on previous research (Bailey et al., 2016), we hypothesized that post-exercise WWI would improve $\dot{\text{V}}$O_2_max and glucose regulation to a similar extent to time-matched exercise, and greater extent than CWI would.

## Research Design and Methods

### Experimental Design

**FIGURE 1:**
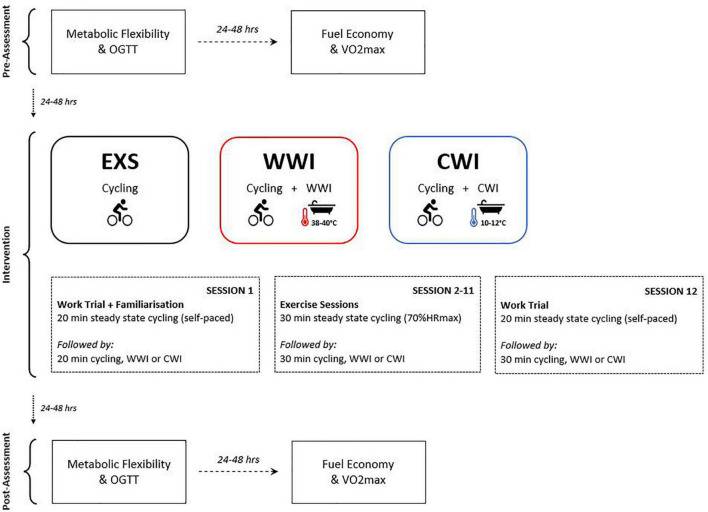
Experimental design. Pre- and post-assessments (OGTT and VO2max) were performed with 24–48 h between each and prior to session one, and after session 12. Sessions 2–11 included the full protocols [EXS (60-min moderate intensity cycling), CWI (30-min cold WI + 30-min moderate intensity cycling) or WWI (30-min warm WI + 30-min moderate intensity cycling)]. A 20-min work trial was completed in session one and 12 followed by EXS, CWI or WWI (time-matched). Participants completed two out of three interventions with a 4-week washout separating each intervention.

A partial-crossover trial was used, employing a balanced, incomplete block design whereby participants were randomized to complete two of three training interventions (12 training sessions over 14–16 days, with 4 week washout between, Figure 1): (i) EXS—60 min of steady state cycling at 70% of maximum heart rate (HRmax) (ii) WWI—30 min of steady state cycling at 70% HRmax followed by 30-min warm water (∼39°C) immersion of the lower limbs, up to umbilicus, and/or iii) CWI—30 min of steady state cycling at 70% HRmax followed by 30 min cold water (∼11°C) immersion of the lower limbs, up to the umbilicus. Prior to session one, all participants completed a non-blinded familiarization cycling work trial to familiarize them to the ergometer (Wattbike) and the 20-min duration. To measure changes in performance, each participant completed a blinded 20-min cycling work trial during the first and last training sessions (instead of continuous cycling). The work trial protocol is outlined below. For each intervention, post-assessments were completed 24 h (OGTT) and 48 h ($\dot{\text{V}}$*O_2_ max*) following the final training session.

### Participants

**FIGURE 2:**
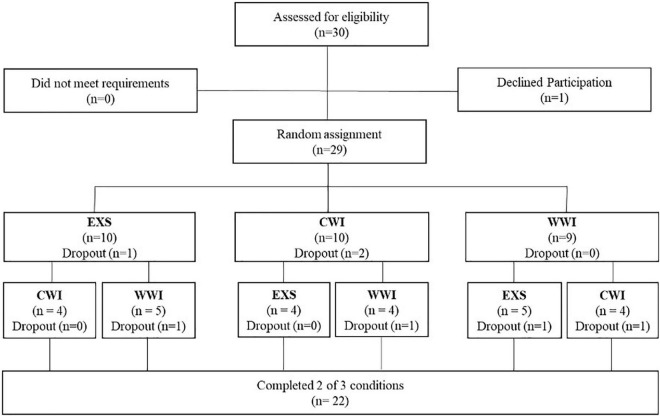
Study flow diagram. Partial randomized crossover trial where participants completed two of three training interventions (each 12 training sessions); 60 min of continuous exercise or 30 min of exercise followed by either 30 min of warm water (∼38–40°C) or cold water (∼10–12°C) immersion to the umbilicus.

Thirty healthy adults were recruited to complete two of three interventions (Figure 2). Twenty two (age = 25 ± 5 y, BMI 23 ± 3 kg/m^2^, 11 females) completed both interventions (EXS: *n* = 17, CWI: *n* = 15, WWI: *n* = 16) and were included in analyses. The eight participants who did not complete both interventions stated time commitment, relocation, intolerance to exercise/cold, surgery or traveling as reasons for dropping out. Participants were recreationally active (participating in physical activity on ∼3 days per week but not in any formal exercise training program). Prospective participants were excluded if they had any previously diagnosed health conditions, smoked (in previous 3 years), were pregnant or had any injury preventing participation. Written informed consent was provided by all participants who were screened for contraindications to exercise using “The Physical Activity Readiness Questionnaire” at an initial consultation. Research protocols were approved by the UOW Human Research Ethics Committee (Ethics Number: 2018/322. Approval Date: 07/08/2018).

#### Training Protocol

For each intervention, participants completed 12 sessions over 14–16 days, with a 4 week washout between (Figure 1). The 2 week duration was chosen to investigate the effects of short-term training whilst minimizing any potential confounding effect of changes in body composition. Several studies have shown benefits in cardiometabolic health after a similar exercise intervention period (Little et al., 2011; Reynolds et al., 2016). All twelve training sessions were supervised by an Accredited Exercise Physiologist. Blood pressure was taken before and after each training session, with heart rate measured via telemetry (Polar Electro, Kempele, Finland) and Rating of Perceived Exertion (RPE) recorded at 5-min intervals throughout each training session.

The first session of the EXS intervention involved 50 min of cycling only, with the first session of WI including 30 min of cycling followed by 20 min of immersion in either cold or warm water. Thereafter, each exercise training session involved continuous cycling at an intensity of 70% of HRmax (assessed via initial $\dot{\text{V}}$*O_2_max* test) for either 30 min (CWI and WWI) or 60 min (EXS) with WI interventions including 30 min of immersion so that all protocols were 60 min in total. All exercise training and water immersion interventions occurred in a room with an ambient 23°C temperature. Participants were able to drink water *ad libitum* during all interventions.

### Water Immersion Interventions

The CWI and WWI interventions involved 30 min cycling at 70% HRmax followed by water immersion for 30 min. Participants sat with their legs fully extended, immersed in water up to their umbilicus between 10 and 12°C (CWI) or 38–40°C (WWI), i.e., temperatures routinely used in water immersion therapy for healthy and clinical populations (Versey et al., 2013). Water temperature was monitored (via a calibrated mercury thermometer) before and throughout immersion to ensure correct temperature was maintained. Hot water/ice was added to the bath and an external tap allowed removal of volume when necessary to maintaining correct immersion temperature and depth. Core temperature (auditory canal, using a Braun ThermoScan), thermal sensation and discomfort scales (Gagge et al., 1967) were measured every 5 min during immersion.

#### Pre- and Post-assessments

##### $\dot{\text{V}}$O_2_max

Participants fasted for 3 h prior to completing two stages of submaximal cycling, at a prescribed absolute and relative intensity, to assess metabolic flexibility (the ability to adapt substrate utilization to substrate availability; Aucouturier et al., 2011), immediately followed by a ramp cycle test to volitional exhaustion for the measurement of $\dot{\text{V}}$O_2_max. Stage one involved 10 min cycling at 50 W for females and 70 W for males (absolute: elicits a low-intensity response of approximately 30–40% of HRmax). In stage two, participants exercised for 10 min at 70% of predicted HRmax (220-age). Indirect Calorimetry (Gas Exchange via Parvo Medic’s TrueOne^*TM*^ 2400) was used to measure participants’ energy use [oxygen uptake ($\dot{\text{V}}$O_2_), expired carbon dioxide ($\dot{\text{V}}$CO_2_)], and substrate utilization (carbohydrate/fat oxidation) (Péronnet and Massicotte, 1991). During the ramp stage, the load was increased by 35 W at 1-min intervals until volitional exhaustion. During the last 30 s of each stage, heart rate, via telemetry and RPE (Borg 6–20 scale; Borg, 1982) were recorded. HRmax was recorded at the completion of the ramp stage.

### Work Trial

Participants completed a self-paced 20-min work trial on an air-braked ergometer (Wattbike, Nottingham, United Kingdom). Participants were encouraged to complete the maximum distance possible, with only their cadence and time used as feedback during the test. Heart rate, distance (km) and power (watts) were recorded at 5-min intervals. A minimum of 48 h separated the $\dot{\text{V}}$O_2_max and work trial sessions and the exercise training interventions.

### Resting Metabolic Rate and Oral Glucose Tolerance Test

Participants fasted for 10 h before test initiation. RMR was measured using a Parvo Metabolic Cart, with a canopy hood to measure rates of $\dot{\text{V}}$CO_2_, $\dot{\text{V}}$O_2_ and RER. Participants were instructed to minimize activity prior to arrival at the laboratory. Following 10 min of supine rest, 20-min RMR data were collected with the final 10 min used for analyses. The same protocol was used for all comparison time points.

Fasting capillary glucose concentration was then measured using a standard lancing device and a HemoCue glucose analyzer (HemoCue Glucose 201 RT glucose; HemoCue AB, Sweden). A standard 75 g glucose tolerance drink was consumed, then RMR was measured for a further 20 min at 30- and 60-min post-consumption. Energy use ($\dot{\text{V}}$O_2_) and fuel utilization (CHO and fat oxidation, assuming minimal protein contribution) (Péronnet and Massicotte, 1991) were calculated at each time point. Capillary glucose concentration was measured 30, 60, 90, and 120 min post-prandially (PP).

#### Data Analysis

Data were first assessed for normality using Q-Q plots, histograms and the Shapiro-Wilk test. After outliers were removed (all data points for that measurement) using the default value of ± 3 standard deviations in SPSS, all assumption of normality conditions were met. The study was powered on effect size change in $\dot{\text{V}}$O_2_max (Bailey et al., 2016) and a linear mixed model (with time × intervention interaction, and main effects of time) was used to compare outcomes between interventions. To determine differences between interventions, *post hoc* tukey tests were performed following a significant interaction. Significance was set at *P* < 0.05. A statistician from UOW was consulted in the study design, performed the blocked randomization and assisted with statistical analyses. 95% CI were calculated using SPSS, and Cohen’s *d* were calculated for effect size [Cohen’s d 0.2–0.5 (small); 0.5–0.8 (moderate); 0.8 + (large)].

## Results

**FIGURE 3:**
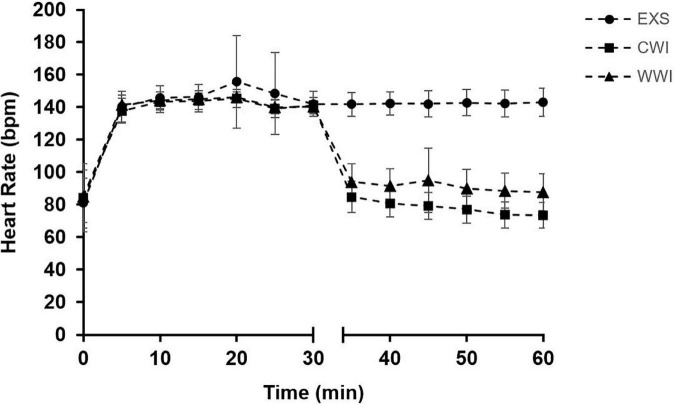
Mean heart rate [HR (bpm)] during time-matched exercise [EXS (*n* = 16)], post-exercise Cold Water Immersion [CWI (*n* = 14)] and post-exercise Warm Water Immersion [WWI (*n* = 14)]. After 30 min of cycling, CWI and WWI began 30 min of water immersion (indicated by gap in *x*-axis). HR was not significantly different between interventions from 0 to 30 min but was significantly higher with EXS than WI (*p* < 0.001) from 0 to 60 min. HR was significantly higher during the 30 min of WWI when compared to CWI (*p* = 0.002).

All participants completed the 12 sessions in 14–16 days, with no more than two consecutive days between training sessions. HR profiles for the three interventions are shown in Figure 3. HR was not different between interventions for the first 30 min of cycling but was significantly higher for the total 60 min during EXS (143 ± 19 bpm) compared to CWI (114 ± 30 bpm) and WWI (121 ± 27 bpm) (interaction *p* < 0.001). HR was significantly higher during WWI than CWI (*p* = 0.002). RPE did not differ between interventions (EXS: 11 ± 1, CWI: 11 ± 1, and WWI: 11 ± 1, interaction *p* = 0.986). On average, auditory canal temperature was not different during the 30 min of WI between WWI (37.4 ± 1.3°C) and CWI (37.5 ± 1.3°C, interaction *p* = 0.931). As expected, thermal sensation differed between CWI (“cold”; 4.0 ± 0.3) and WWI (“slightly warm”; 8.0 ± 0.3) (interaction *p* < 0.001). Thermal discomfort was greater with CWI (“slightly uncomfortable”; 2.0 ± 0.2) compared to WWI (“comfortable”; 1.0 ± 0.1) (interaction *p* = 0.052).

### $\dot{\text{V}}$O_2_max

**FIGURE 4:**
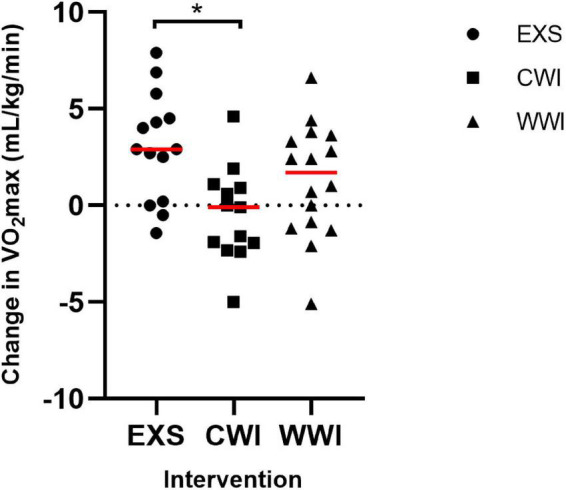
Change in cardiorespiratory fitness ($\dot{\text{V}}$O_2_max [mL/kg/min]) following 12 sessions of Exercise [EXS (*n* = 14)], post-exercise Cold Water Immersion [CWI (*n* = 14)] and post-exercise Warm Water Immersion [WWI (*n* = 16)]. *Indicates significant difference between EXS and CWI (*post hoc*, *p* = 0.002), following significant interaction (*p* = 0.004).

The change in $\dot{\text{V}}$O_2_max differed between interventions [interaction *p* = 0.004, Figure 4; EXS (Pre: 39.9 ± 7.4 mL/kg/min to Post: 43.6 ± 6.4 mL/kg/min, *p* = 0.001), CWI (Pre: 41.8 ± 7.7 mL/kg/min to Post: 41.3 ± 8.1 mL/kg/min, *p* = 0.495), WWI (Pre: 40.0 ± 8.9 mL/kg/min to Post: 41.3 ± 9.0 mL/kg/min, *p* = 0.103)]. EXS significantly increased $\dot{\text{V}}$O_2_max (large effect) by 3.2 mL/kg/min more than CWI (CI: 1.1, 5.3 mL/kg/min, *d* = 1.36), but not significantly more than WWI (CI: −0.4, 3.9 mL/kg/min, *d* = 0.73).

### Work Trial

Distance (time effect *p* = 0.048) and power (time effect *p* = 0.008) increased for the 20-min work trial, by an average 380 m and 20 W, respectively, with no difference between interventions [interactions Distance (*p* = 0.692), Power (*p* = 0.812), Table 1].

**TABLE 1:** Total distance traveled, average heart rate (HR) and power during the 20-min cycle ergometer work trial.

	**Distance (km)**			**HR (bpm)**			**Power (watts)**		
	**Pre**	**Post**	**Change**	**Pre**	**Post**	**Change**	**Pre**	**Post**	**Change**
EXS	11.4 ± 0.93	12.0 ± 0.97	0.58^*^ ± 0.64	168 ± 23	176 ± 11	8 ± 18	160 ± 41	187 ± 34	27^*^ ± 22
CWI	11.2 ± 0.65	11.6 ± 0.66	0.43^*^ ± 0.48	166 ± 20	172 ± 13	6 ± 10	152 ± 24	171 ± 28	19^*^ ± 20
WWI	11.4 ± 0.96	11.5 ± 1.1	0.14^*^ ± 0.46	170 ± 8	170 ± 10	0.36 ± 9	160 ± 35	175 ± 45	14^*^ ± 14

Values are means ± SD. Data are mean of pre and post-intervention work trial for each participant [Exercise (EXS n = 15)], Cold Water Immersion (CWI n = 14), Warm Water Immersion [WWI, n = 14)]. No significant difference between interventions. *Indicates significant main effect of time.

### Glucose Tolerance (Oral Glucose Tolerance Test)

**FIGURE 5:**
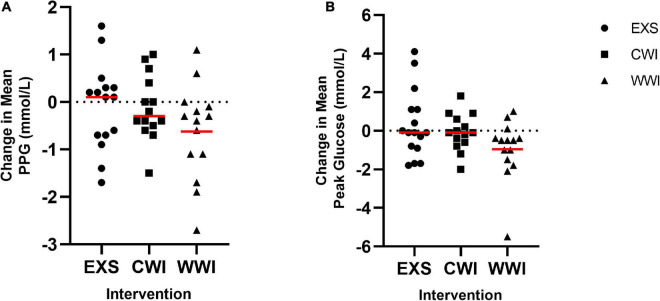
**(A)** Change in post-prandial Glucose (PPG) after 12 sessions of time-matched exercise [EXS (*n* = 15)], post-exercise Cold Water Immersion [CWI (*n* = 14)] and post-exercise Warm Water Immersion [WWI (*n* = 14)]. No significant interaction effect. **(B)** Change in Peak PPG after 12 sessions of EXS (*n* = 16), CWI (*n* = 14) and WWI (*n* = 14). No significant interaction effect.

Fasting (interaction *p* = 0.769), mean (interaction 0.193) and peak post-prandial glucose (PPG) (interaction = 0.149) were not significantly different between interventions. The change in mean PPG is shown in Figure 5A. Mean PPG changed by + 0.1 mmol/L following EXS (CI−0.5, 0.9 mmol/L; *d* = 0.19), −0.2 mmol/L following CWI (CI−0.5, 0.3 mmol/L; *d* = 0) and −0.6 mmol/L following WWI (CI−1.2, −0.03 mmol/L; *d* = 0.62). Peak PPG showed a small change after EXS (CI −0.6, 1.2 mmol/L, *d* = 0.21) and no change after CWI (CI −0.6, 0.6 mmol/L; *d* = 0.00), but WWI was found to moderately decrease peak PPG by −1.0 mmol/L (CI −1.9, −0.5 mmol/L; *d* = 0.63). The change in Peak PPG is shown in Figure 5B.

### Metabolic Flexibility

#### Fuel Utilization Glucose Challenge Oral Glucose Tolerance Test

Fasting (*p* = 0.67) and post-prandial RER (*p* = 0.71) were not significantly different between interventions, nor was fasting (*p* = 0.39) or post-prandial (*p* = 0.40) carbohydrate (CHO) utilization (Table 2).

**TABLE 2:** Fasting and post-prandial (PP) respiratory exchange ratio (RER) and substrate use [Carbohydrate (CHO)], following 12 sessions of time-matched Exercise [EXS (*n* = 15)], post-exercise cold water immersion [CWI (*n* = 14)] and post-exercise warm water immersion [WWI (*n* = 14)] during a 2 h Oral Glucose Tolerance test.

	**Fasting**		**Post-prandial**		**Fasting CHO**		**Post-prandial CHO**		**RER change**		**Fat usage**		**Fat usage**		**Watts at**	
	**RER**		**RER**		**(cal/min)**		**(cal/min)**		**rest to EXS**		**(cal/min)**		**(cal/min)**>		**70% HRmax**	
											**absolute watts**		**relative watts**			
	**Pre**	**Post**	**Pre**	**Post**	**Pre**	**Post**	**Pre**	**Post**	**Pre**	**Post**	**Pre**	**Post**	**Pre**	**Post**	**Pre**	**Post**
EXS	0.84 ± 0.07	0.86 ± 0.09	0.90 ± 0.06	0.88 ± 0.04	56.55 ± 29.49	63.22 ± 30.60	84.88 ± 29.13	79.45 ± 20.96	−0.02 ± 0.13	0.01 ± 0.09	246.75 ± 111.24	238.95 ± 65.44	166.95 ± 108.63	169.93 ± 80.45	99 ± 40	113 ± 42
CWI	0.82 ± 0.05	0.82 ± 0.05	0.88 ± 0.06	0.86 ± 0.05	47.60 ± 23.35	47.98 ± 30.39	77.53 ± 35.03	67.60 ± 34.21	0.01 ± 0.06	0.04 ± 0.14	266.97 ± 126.55	214.62 ± 78.30	198.30 ± 159.84	168.25 ± 121.03	106 ± 28	110 ± 31
WWI	0.88 ± 0.11	0.85 ± 0.07	0.91 ± 0.06	0.88 ± 0.06	71.52 ± 39.65	62.03 ± 31.81	99.17 ± 29.62	74.88 ± 28.54	0.01 ± 0.11	−0.01 ± 0.08	202.77 ± 56.60	215.83 ± 85.33	98.64 ± 100.41	158.52 ± 104.61	95 ± 46	113 ± 35

*Fat utilization, at an absolute (70 Watts Males/50 Watts Females) and relative watts (70% HRmax) was measured during submaximal exercise. Values are Mean ± SD. No significant difference between interventions.*

#### Fuel Utilization Submaximal Exercise

Change in RER from rest to exercise was not significantly different between interventions (interaction *p* = 0.676) (Table 2). Fat utilization at an absolute (interaction *p* = 0.270) and relative power (interaction *p* = 0.515) was not significantly different between interventions, nor across time (time effect *p* = 0.687). Power output at 70% of HRmax was not significantly different between interventions (interaction *p* = 0.443) nor across time (*p* = 0.158).

## Discussion

This study investigated whether substituting the final 30 min of each exercise bout with warm or cold water immersion could provide similar or greater benefits for cardiometabolic health and exercise performance. The main finding was that exercise training alone (EXS) results in the greatest increase in cardiovascular fitness ($\dot{\text{V}}$O_2_max), being significantly greater than the change following 2-weeks of post-exercise CWI but not post-exercise WWI. All short-term interventions improved performance related indices such as power output and distance attained during a 20 min work trial (albeit this study did not include a non-intervention control). WWI was also perceived as thermally comfortable, which may further facilitate its efficacy as an adjunct to exercise training, allowing for less exercise whilst still achieving cardiometabolic health benefits in people who cannot/will not perform sufficient volumes of exercise. Although the changes in metabolic indices were not significantly different between interventions, future research is needed given the non-significant but moderate decrease in peak post-prandial glucose after WWI. WWI used alone has previously been shown to improve glucose control (Hooper, 1999; Hoekstra et al., 2018), and thus warrants further research as an adjunct to exercise for improving glucose tolerance in those at risk. Together, our study adds to the current literature on hydrotherapy and highlights post-exercise WWI as a potential alternative to longer duration exercise. Given similar benefits to longer duration exercise, post-exercise WWI has important implications for those who are either unable or disinclined to exercise for 60 min, such as those with low exercise tolerance, chronic obstructive pulmonary disease, congestive heart failure, cardiomyopathy, peripheral vascular disease, obesity and Type 2 Diabetes.

### Cardiorespiratory Fitness ($\dot{\text{V}}$O_2_max)

The benefits of ∼300 min/week of exercise training (physical activity guidelines) on fitness are well known. The present study found that, although $\dot{\text{V}}$O_2_max improved to a greater extent with EXS alone when compared to post-exercise CWI, there were no significant differences between EXS alone and when 30 min of exercise was substituted with 30 min of post-exercise WWI. This suggests WWI can be a partial substitute to exercise for achieving gains in fitness. Bailey et al. (2016) found comparable improvements in cardiovascular fitness after 30 min of WWI alone vs. time-matched moderate-intensity cycling, whereas we have shown here that substituting the second half of an exercise session with WWI also provides comparable benefits. In contrast, post-exercise CWI was less effective than EXS alone for improving cardiorespiratory fitness. Prior work by Broatch et al. (2017) also showed CWI did not improve $\dot{\text{V}}$*O*_2_ max or maximal uncoupled respiration (complexes I and II), compared to 6-weeks of exercise training, supporting the work of Yamane et al. (2006, 2015) in showing blunted vascular, endurance and strength effects of CWI. Future research on CWI is needed to determine the mechanisms by which CWI may blunt exercise adaptations (e.g., blood flow, inflammation and cellular metabolism) (Pedersen and Hoffman-Goetz, 2000; Mawhinney et al., 2017; Peake et al., 2017).

This is the first study to compare the effects of post-exercise WWI, CWI, and EXS. The duration and intensity of our interventions are in line with current physical activity recommendations that suggest 150–300 min of moderate intensity exercise per week for improvements in cardiorespiratory fitness (Australian Government Department of Health [AGDH], 2020). The larger increase in cardiorespiratory fitness with EXS than CWI presumably reflects at least partly a greater cardiovascular stimulus. Thomas et al. (2016) previously showed that WWI and exercise increase muscle temperature, limb blood flow and antegrade shear stress, with the increases following WWI being of a greater extent compared to exercise. Indeed, many key adaptations to exercise training are stimulated by elevations in muscle and body temperature, resulting in increases in blood flow and volume, metabolic rate, and the regulation of gene expression and signaling molecules i.e., for mitochondrial biogenesis (Thomas et al., 2016). Exercise remains superior for improving cardiovascular fitness, however, for those who are unable to perform larger volumes of exercise, WWI could be used as an alternate option to retain $\dot{\text{V}}$O_2_max or achieve some improvement.

### Exercise Performance Work Trial

Exercise performance, measured as work trial distance and power, improved after all interventions. In contrast, prior research on exercise performance and WI (often used to promote recovery) has shown little to no effect on exercise training adaptations (Broatch et al., 2018). For example, Vaile et al. (2008) found that CWI following exercise led to modest improvements in time trial performance but did not find any benefits with WWI. Likewise, Malta et al. (2021) concluded that aerobic exercise performance, specifically, mean power and time-trial performance (duration) were not adversely effected by regular use of CWI. Our findings that post-exercise CWI and WWI had similar (but no reliably identifiable additive or detrimental) effects for performance indicators compared to time-matched exercise alone, suggest WI could be used as an alternate to exercise alone. We acknowledge that without a 30-min exercise alone intervention, it is unclear whether WI is additive in our study. The aim of this proof-of-concept study was to see if WI could provide comparable effects as an exercise substitute for those who cannot perform sufficient volumes of exercise. Previous research comparing 30–45 min of post-meal exercise has found similar benefits for post-prandial glucose, however, benefit to fitness and exercise performance needs to be further investigated (Bellini et al., 2021).

### Metabolic Health

Continued research on hydrotherapy is needed to determine the impact of WI as an exercise adjunct for enhancing metabolic health. In contrast to Hoekstra et al. (2018), the current study found no intervention effect on fasting blood glucose, mean PPG or metabolic flexibility; despite similar intervention durations. This may be due to differences in water immersion depth (neck) and duration (60 min), which were used by Hoekstra et al. (2018) would likely elicited greater thermal stress and thus adaptation. Despite there being no significant difference between interventions, our WWI results are promising given the on average 1.0 mmol/L change in peak PPG following the short-term WWI intervention; particularly in an apparently healthy population where blood glucose improvements would be expected to be minimal. A 1 mmol/L (∼10%) has been associated with improved glycemic control, reduced risk of diabetes related complications and CVD (Mazzone, 2010; Hrubeniuk et al., 2020). Furthermore, prior studies have shown that WWI interventions improve HbA1c, fasting blood glucose and insulin sensitivity in at risk populations (McCarty et al., 2009; Hooper et al., 2014; Krause et al., 2015). The proposed mechanisms underlying these changes in other studies are an increase in heat shock protein (iHsp72) expression and reduced chronic low-grade inflammation (Hoekstra et al., 2020). Further investigation regarding the intensity of exercise preceding WWI, the required temperature of water and the duration of immersion, in metabolically compromised individuals, is required to better understand how WWI could be used as an adjunct therapy.

## Future Directions

The aim of the present study was to investigate the feasibility and efficacy of post-exercise WI in an apparently healthy population before implementing this approach in clinical populations. In keeping with the physical activity recommendations and to allow translation of results to “free living,” insufficiently active adults, moderate intensity physical activity was chosen for the present study. Further to this, WI involved temperatures and depths that individuals could implement at home in the future. Improvements in work trial performance after all interventions suggest that partial substitution of exercise training, with either WWI or CWI, can improve performance indicators despite the reduced exercise volume. Whilst changes in $\dot{\text{V}}$O_2_max were similar between EXS and post-exercise WWI, CWI did not lead to improvements in cardiorespiratory fitness. Our findings, together with previous work, suggest WWI can be used in clinical populations to enhance their ability to perform physical activity. Adherence to WWI in Akerman et al. (2019) was ∼four times higher than the exercise only group. In the present study, discomfort experienced during CWI was significantly greater than with WWI. Together with the fitness benefits, this comfort indicates post-exercise WWI may be more efficacious than CWI due to the reduced discomfort experienced by participants, thus potentially improving exercise compliance. Future studies should focus on WWI as an adjunct to exercise training for health benefits.

## Strengths and Limitations

A 4 week washout period was implemented, allowing participants to return to baseline for main outcomes of cardiorespiratory fitness and glucose control before beginning their second intervention and to ensure reversibility of training effect (Hecksteden et al., 2018). We also employed 30 min of WI, that together with exercise training is in line with the current physical activity guidelines of 150–300 min per week. Body mass was not a contributing factor and did not change significantly from baseline with any of the interventions; this was by design and thus expected. Ideally, participants would have completed all three trial interventions (complete crossover design), however, the associated time commitment and seasonal impact may have deterred participation and resulted in larger dropouts. Further to this, we acknowledge the limitations of prescribing exercise relative to fixed percentages of HRmax (Jamnick et al., 2020). As mentioned above, a limitation of the present study, was that it did not include a 30-min exercise only intervention, therefore it is unknown whether WI is indeed additive. In addition, because this proof-of-concept study involved young, healthy subjects, this may have contributed to an increased ceiling effect for significant differences in other metabolic outcomes, particularly for glucose tolerance. As such, duration (12 sessions) and intensity (moderate) of intervention may not have been enough to elicit significant differences.

## Conclusion

This study adds to current literature highlighting the therapeutic potential of post-exercise WWI. Findings from this study show that 2-weeks of post-exercise WWI can provide similar benefits for cardiovascular fitness and time trial performance compared to 60-min of EXS alone, whereas EXS was superior to post-exercise CWI for cardiovascular fitness. For people who cannot exercise for the full 60 min, substituting 30 min of the exercise session with WWI provides a promising adjunct to achieve similar positive health outcomes. The combination of WWI and exercise in the present study is noteworthy given WI is not a full substitute for all exercise adaptations (e.g., bone density, calcium handling, neuromuscular junction maintenance, etc.). Results pertaining to metabolic health remain inconclusive and further investigation is required to determine if this could be used as a viable option for individuals with obesity or type 2 diabetes.

## Data Availability Statement

The raw data supporting the conclusions of this article will be made available by the authors, without undue reservation.

## Ethics Statement

The studies involving human participants were reviewed and approved by UOW Human Health and Medical Research Committee. The patients/participants provided their written informed consent to participate in this study.

## Author Contributions

MF, TH, and JC designed the study. BR, CC, TH, and MF conducted the research. BR, CC, and MF analyzed the data. BR composed the manuscript. All authors edited the manuscript and approved the final draft.

## Conflict of Interest

The authors declare that the research was conducted in the absence of any commercial or financial relationships that could be construed as a potential conflict of interest.

## Publisher’s Note

All claims expressed in this article are solely those of the authors and do not necessarily represent those of their affiliated organizations, or those of the publisher, the editors and the reviewers. Any product that may be evaluated in this article, or claim that may be made by its manufacturer, is not guaranteed or endorsed by the publisher.
